# BRIDGE to liver health: implementation of a group telehealth psychoeducational program through shared medical appointments for MASLD management

**DOI:** 10.1186/s12889-024-18865-4

**Published:** 2024-06-07

**Authors:** Nicole Dalal, Lisa Catalli, Sara A. Miller, Simone Madan, Riley Tan, Eliana Agudelo, Danielle Brandman

**Affiliations:** 1 Department of Medicine, University of California San Francisco, San Francisco, CA USA; 2 Division of Gastroenterology and Hepatology, University of California San Francisco, San Francisco, CA USA; 3 Division of General Internal Medicine, Behavioral Medicine Unit, University of California San Francisco, San Francisco, CA USA; 4 Department of Surgery, University of California San Francisco, San Francisco, CA USA; 5 Division of Gastroenterology and Hepatology, Weill Cornell Medicine, New York, NY USA; 6 Center for Liver Disease and Transplantation, New York Presbyterian-Weill Cornell Medicine, 1305 York Ave, Y414, New York, NY 10021 USA

**Keywords:** Advanced practice providers, Behavior modification, MASLD, Shared medical appointment, Telemedicine

## Abstract

**Background:**

Metabolic dysfunction-associated steatotic liver disease (MASLD) represents a significantly costly and increasingly prevalent disease, with treatment focused on lifestyle intervention. Integrating education and behavioral health into clinical care offers opportunities to engage and empower patients to prevent progression of liver disease. We describe the design and implementation of Behavioral Resources and Intervention through Digital Group Education (BRIDGE), a 6-session group telehealth program led by advanced practice providers (APPs) in 90-min shared medical appointments (SMAs) with small groups of MASLD patients in an academic outpatient hepatology clinic. The program contains multi-component group interventions, with didactic education and behavioral coaching, while leveraging peer-based learning and support.

**Methods:**

A mixed-methods exploratory pilot study was conducted. Feasibility and acceptability of the clinical intervention were assessed by tracking recruitment, attendance, and retention of BRIDGE participants, patient interviews, and debriefing of clinician and staff views of the clinical program. Implementation metrics included program development time, workflow and scheduling logistics, and billing compliance for sustainability. Finally, patient parameters including changes in liver enzymes, FIB-4, weight, and BMI from pre- to post-BRIDGE were retrospectively analyzed.

**Results:**

We included 57 participants (median age 57, interquartile range (IQR) 50 – 65 years), 38 (67%) female, 38 (67%) white, and 40% had public insurance. Thirty-three (58%) participants completed all six sessions, while 43 (75%) attended at least five sessions. Patients who completed all sessions were older (median age 61 vs 53.5; *p* = 0.01). Gender, race/ethnicity, and insurance type were not significantly associated with missed sessions, and patients had similar rates of completion regardless of weight, BMI, or stage of liver disease. Barriers to completion included personal illness, family reasons, work commitments, or insurance issues. Prior to BRIDGE, median BMI was 31.9 (SD 29 – 36), with a median weight loss of 2 pounds (IQR -2 – 6) after BRIDGE.

**Conclusion:**

The BRIDGE telehealth SMA program was feasible, well-attended, and positively reviewed. This pilot study informs future iterations of program development and evaluation of outcome measures.

## Background

Metabolic dysfunction-associated steatotic liver disease (MASLD), defined as hepatic steatosis with one cardiometabolic risk factor and no other clear etiology of liver dysfunction [1], is the most common chronic liver disease, affecting 25% of individuals worldwide and 30% of the US population, with rapidly increasing prevalence [2]. MASLD is also a leading indication for liver transplantation [3]. However, the impact of MASLD and its associated comorbidities extend beyond the liver, with morbidity and mortality in these patients dominated by cardiovascular events and cancer [4–9]. The healthcare financial burden of MASLD in the US is also significant, with estimates ranging from direct annual medical costs of $103 billion dollars per year (approximately $1,613 per patient) [6] to higher projections among privately insured patients – accounting for $7,804 annually for new diagnoses of MASLD versus $3,789 for long-term management [10].

No liver-directed pharmacotherapy for MASLD has thus far been approved by regulatory agencies for widespread use. The mainstay of therapy for MASLD relies upon empowering patients to adopt and implement lifestyle modifications to optimize cardiometabolic health and sustained weight loss [11, 12]. Weight loss interventions have been associated with improved liver biomarkers as well as decreased radiologic and histologic evidence of liver steatosis [13]. A multi-disciplinary approach provides the best chance for success in reducing liver and cardiovascular morbidity and mortality [11].

The US Preventative Services Task Force (USPSTF) recommends referring patients with obesity to multicomponent behavioral interventions [14]. Group-based interventions that employ motivational interviewing (MI) and provide personalized information with feedback on specific dietary/physical activity goals offer effective approaches [15, 16]. Several studies have demonstrated that psychoeducational community/clinic-based group programs provide effective behavioral counseling [17–24]. The Diabetes Prevention Program (DPP) is a community-based group intervention that integrates behavioral education aimed at providing accountability for individuals with pre-diabetes to promote sufficient weight loss to delay the onset of type 2 diabetes [25, 26]. Group lifestyle interventions based on the DPP model have been shown to increase motivation to lose weight [25–27] and have been successfully modified for in-person and telehealth primary and obesity care [27–29].

A Shared Medical Appointment (SMA) care delivery model can bring group lifestyle interventions into mainstream clinical care by bringing patients with similar health problems together in concurrent extended clinic visits with one or more clinicians. SMA sessions are comprehensive, involving an educational component and facilitated peer interaction around self-management and empowerment [30, 31]. SMAs are often led by teams of licensed clinicians (ie physicians, nurses, pharmacist, dieticians, mental health providers) [30, 31], yet under-resourced settings may not have access to a multidisciplinary team. Feasibility studies have shown that the cost and attendance of group programs can be optimized through telehealth by removing structural or economic barriers to care, such as eliminating travel time to in-person visits and allowing for more frequent visits [32–34].

Advanced Practice Providers (APPs) have been shown to successfully deliver behavioral change interventions, including for patients with heart failure [35] opioid use disorder [36], and diabetes [37, 38]. Given the roots of the APP professions in health promotion, they may be best positioned to promote behavior change for MASLD patients [39]. To this end, we studied the feasibility and acceptability of an APP-led longitudinal SMA telehealth program focused on educating and empowering patients with MASLD in an academic hepatology practice setting.

## Methods

This mixed-methods exploratory pilot study was used to evaluate the feasibility and acceptability of our SMA telehealth program, a videoconferencing-enabled group psychoeducational program entitled Behavioral Resources and Intervention through Digital Group Education (BRIDGE). Relative aspects of the Consolidated Standards of Reporting Trials for pilot and feasibility trials (CONSORT) [40, 41] were followed in preparation of the manuscript. Feasibility was assessed via metrics of patient recruitment, attendance and retention, along with input from APPs and medical staff regarding implementation and sustainability from a billing/coding standpoint and workflow/scheduling logistics. Acceptability was based on patient and staff feedback regarding their perceptions of the program. Finally, patient metrics were collected to assess preliminary outcomes after participation in BRIDGE. Below, we describe the development, implementation, and assessment of the external pilot study of BRIDGE.

### Program development

We developed a patient-centered program focusing on education and evidence-based behavioral strategies in weight management while harnessing MI to engage patients in collaborative dialogue. To guide program development, a needs assessment questionnaire with 8 multiple choice questions was sent to a large cohort of patients with MASLD who received outpatient Hepatology Care at the the University of California at San Francisco (UCSF) via our institution’s web-based survey tool (Qualtrics). Patients were queried to gauge interest in participating in a group telehealth program, understand curricular learning needs, and determine a preferred frequency of sessions. Out of the 220 surveys distributed, 21 responses were received, and we used this input to guide program development.

We performed a comprehensive literature review on multi-component behavioral interventions in weight management programs to guide the theoretical framework and structure of the BRIDGE program. The behavioral interventions throughout the entire program integrated MI principles as described in *Motivational Interviewing in Health Care* [42] and *Motivational Interviewing in Groups* [19], the transtheoretical model of behavior change [43] and cognitive behavioral therapy strategies (antecedent-behavior-consequence, goal setting, problem solving and self-monitoring) [17, 28, 44, 45]. The structure of the BRIDGE program was guided by utilizing components from the National Diabetes Prevention Program [46], the MoDEL-IBT Program [27], and the UCSF Survivorship Wellness Program [47].

Subsequently, input from our team, initially consisting of one physician, two hepatology Nurse Practitioners (NPs), and a consulting psychologist collaborated in the design of a series of six psychoeducational sessions for groups of five patients. Further refinement of the content of each session was guided by a weight management psychologist with contributions from a registered dietitian (for educational materials on nutrition).The sessions were entitled “Introduction to BRIDGE and MASLD,” “Improving Health Outcomes in MASLD,” Motivation and Making Change,” “Eating Habits for a Healthy Liver,” “Physical Activity for a Healthy Liver,” and “Stress Management.” The didactics for stress management and creation of a “goal tracker” were derived from the work of the psycho-oncologist on an interdisciplinary survivorship wellness program for cancer survivors at our institution [47]. Both NPs had prior palliative care training (through Vital Talk, https://www.vitaltalk.org/), and one had received specific training in obesity and lifestyle medicine. This interdisciplinary team met frequently (at least quarterly) to further refine the content across several iterations of the program, to discuss challenging cases, and to share successes. Throughout the program duration, BRIDGE group leaders engaged in self-directed learning and continuing education courses in MI and cognitive behavioral strategies for lifestyle interventions. A physician assistant (PA) was trained as a group leader through shadowing and co-leading sessions.

### Patient selection

BRIDGE was offered to adult patients followed in the UCSF hepatology clinic with a diagnosis of MASLD by non-invasive testing or biopsy, at any stage of liver fibrosis. Patients with clinically significant portal hypertension were excluded from the BRIDGE cohorts, as their educational needs significantly varied from the rest of the cohort. Furthermore, patients who had received a liver transplant were excluded from this initial cohort. Certain patients in our MASLD clinic were not approached to participate in BRIDGE, including those with lack of access to internet/electronic devices, untreated mental illness, ongoing alcohol misuse or AUD, decompensated liver disease, or who indicated discomfort with shared medical appointments with other patients. Of the 119 patients who were invited to participate between 11/2020–1/2022, 57 participated (47.9%). Groups were divided into adults > 55 years old and ≤ 55 years old based on clinical decision-making and patient preference to be grouped with others of similar ages for shared visits to foster closer connection and engagement. Each group was heterogeneous with regards to gender and age. Additionally, BRIDGE content was translated into Spanish; one cohort was primarily Spanish-speaking, and their sessions were led by a certified bilingual English/Spanish-speaking APP.

### Logistics/operations

Once patients were referred to the program by their hepatology provider, medical assistants (MAs) called patients to facilitate enrollment and were provided a script to introduce the program in a standardized way. A signed or verbal group confidentiality consent was documented prior to participating in the program, due to the potential for sharing of medical information among group participants. Patients were advised that they would be billed for each session as a 1:1 standard clinic visit, with charges made according to their respective insurance coverage plans. After confirming interest in participation, MAs obtained a prior authorization for all six sessions from the patient’s insurance carrier for Current Procedural Terminology (CPT) code 99213 (established patient visit, 20–29 min) and added the patient to the group visit schedule. MAs also sent group confidentiality consent forms to participants, and occasionally called to assist with connectivity issues. Before each session, participants were also sent slides and educational handouts that would be reviewed in detail during the sessions. MAs spent approximately 13 min per patient in activities that included enrollment, scheduling, and prior authorization.

Hepatology APPs led the six sessions every other week, which were comprised of a formal didactic component with interactive group discussions/activities before and after the didactics to build group cohesion and help patients develop strategies to achieve and sustain weight loss. Each slide contained standardized notes to follow and maintain fidelity of the program. The group discussions focused on determining readiness for behavior change, learning how to self-monitor habits, and planning for challenges with adhering to implemented changes. Patients were instructed on use of a goal tracker to identify and follow their personal behavior change goals. Furthermore, participants were educated on the utilization of a food diary using the modality of their preference, such as pen and paper, web-based, and mobile application-based.

The first session began with the APP facilitating an icebreaker activity to engage members, followed by a review of program goals, request to maintain confidentiality, and best practices for group telehealth participation (muting microphone during didactics, saving questions for the end of the didactics, and using the group chat function). Most importantly, the APP established group norms by asking participants to support other group members rather than advise, unless explicitly asked. Each subsequent session was started with unstructured conversation to share “what went well, or what were the challenges?” This component of the sessions became progressively less structured, to allow for open conversation. A 15–20-min real-time didactic presentation was performed by the APP leader, followed by questions and answers, then a group activity, on average lasting a total of 45–60 min. Sessions typically concluded with five-minute one-on-one sessions between each group participant and the APP leader in a separate breakout room, while others participated in a group activity and discussion for a total of 25–30 min. The duration of each session was on average 90 min.

### Program assessment

Institutional Review Board (IRB) approval was obtained for this biomedical research including medical records review, health care or health outcomes-related activities and data analysis. The study received a waiver of consent by the IRB due to the study meeting criteria for minimal risk. The UCSF Committee on Human Research approved the study, and this work was conducted in accordance with the Declaration of Helsinki and Istanbul.

A post-program survey was sent via our institution’s web-based survey tool (Qualtrics) to the first 2 cohorts of patients who participated in the pilot study from November 2020 to May 2021, and feedback was also collected from APPs and medical staff regarding their experiences in order to assess program feasibility and acceptability.

Retrospective record review of 57 BRIDGE participants from November 2020 to January 2022 included de-identified clinical data on history of diabetes, hypertension, hyperlipidemia, obesity, stage of liver disease, BMI, alcohol intake, AST, ALT, total bilirubin, alkaline phosphatase, albumin, and platelet count. Data was stored in a password protected file on an encrypted driver. Patient identifiers were stored separately. We evaluated changes in liver enzymes, FIB-4, weight, and BMI from pre- to post-BRIDGE. We further assessed differences in patient characteristics associated with completion of the entire BRIDGE program.

## Results

Between November 2020 to January 2022, 57 individuals participated in BRIDGE (Table 1). Median age was 57, with interquartile range (IQR, 50 – 65 years); 38 (67%) were female, 38 (67%) were white, 44 (77%) were not Hispanic/Latino, and 40% had public insurance (12% Medicaid [MediCal], 28% Medicare). Average weight at enrollment was 193.3 pounds (standard deviation [SD] 172 – 228), with BMI 31.9 (SD 29 – 36). The majority of patients (67%) had early stage NAFLD (fibrosis 0–1) at enrollment. Three patients were primarily Spanish speaking.

**Table 1:** Baseline characteristics of BRIDGE participants (*n* = 57)

Patient characteristics	Value
**Median Age (years, IQR)**	57 (50–65)
**Gender (number, %)**	
Male	19 (33%)
Female	38 (67%)
**Race (number, %)**	
White	38 (67%)
Black	1 (2%)
Asian	10 (17%)
Other Pacific Islander	1 (2%)
Other	7 (12%)
**Ethnicity (number, %)**	
Hispanic/Latino	13 (23%)
Not Hispanic/Latino	44 (77%)
**Insurance Status (number, %)**	
Medicare	16 (28%)
Medicaid	7 (12%)
Private	34 (60%)
**Weight at enrollment (pounds, SD)**	193.3 (172–228)
**BMI at enrollment (SD)**	31.9 (29–36)
**Fibrosis stage at enrollment**	
0–1	38 (67%)
2	8 (14%)
3	5 (9%)
4	6 (10%)
**Participants who missed sessions**	
No sessions missed	33 (58%)
One or more sessions missed	24 (42%)
One session missed	10 (18%)
More than one session missed	14 (24%)
**Reason for missed sessions**	
Family commitment	2 (8%)
Personal illness	3 (13%)
Insurance/copay issues	2 (8%)
Work	2 (8%)
Other personal reasons/unknown	15 (63%)

Thirty-three (58%) participants attended all six sessions, and 43 (75%) attended at least five sessions. Patients who attended all sessions were older (median age 61 vs 53.5; *p* = 0.01). Gender, race/ethnicity, and insurance type (including public insurance) were not significantly associated with missed sessions. Patients appeared to have similar rates of program completion regardless of weight, BMI, or fibrosis stage (Table 2). Though not statistically significant, women (*p* = 0.09) and Hispanic patients (*p* = 0.11) were more likely to have missed sessions. Reported reasons for missed sessions included personal work or illness, family reasons, work commitments, or insurance issues. However, insurance type (*p* = 0.57) and baseline pre- and post-BRIDGE BMI (*p* = 0.52) and baseline liver enzymes (ALT, *p* = 0.70; AST, *p* = 0.57) did not impact program completion rates. Patients with cirrhosis had the highest rate of program completion (83%). There was no difference in amount of weight loss or BMI change whether patients did or did not attend all BRIDGE sessions.

**Table 2:** Patient factors associated with completion of the BRIDGE program

	**BRIDGE Completion Status**		
	Completed	Not Completed	*p* value
**Median Age (years; IQR)**	61 (54–67)	53.5 (44–60)	0.01
**Gender (n, % female)**	19 (58%)	19 (79%)	0.09
**Race**			0.26
White	23 (61%)	15 (39%)	
Black	0 (0%)	1 (100%)	
Asian	7 (70%)	3 (30%)	
Other Pacific Islander	1 (100%)	0 (0%)	
Other	2 (29%)	5 (71%)	
**Ethnicity**			0.11
Hispanic/Latino	5 (38%)	8 (62%)	
Not Hispanic/Latino	28 (64%)	16 (36%)	
**Insurance Type**			0.57
Medicare	11 (69%)	5 (31%)	
Medicaid	4 (57%)	3 (43%)	
Private	18 (53%)	16 (47%)	
**Fibrosis stage at time of BRIDGE enrollment**			0.10
0–1	24 (63%)	14 (37%)	
2	2 (25%)	6 (75%)	
3	2 (40%)	3 (60%)	
4	5 (83%)	1 (17%)	
**Advanced Fibrosis**			0.67
No	26 (57%)	20 (43%)	
Yes	7 (64%)	4 (36%)	

### Feasibility/acceptability

**Fig. 1:**
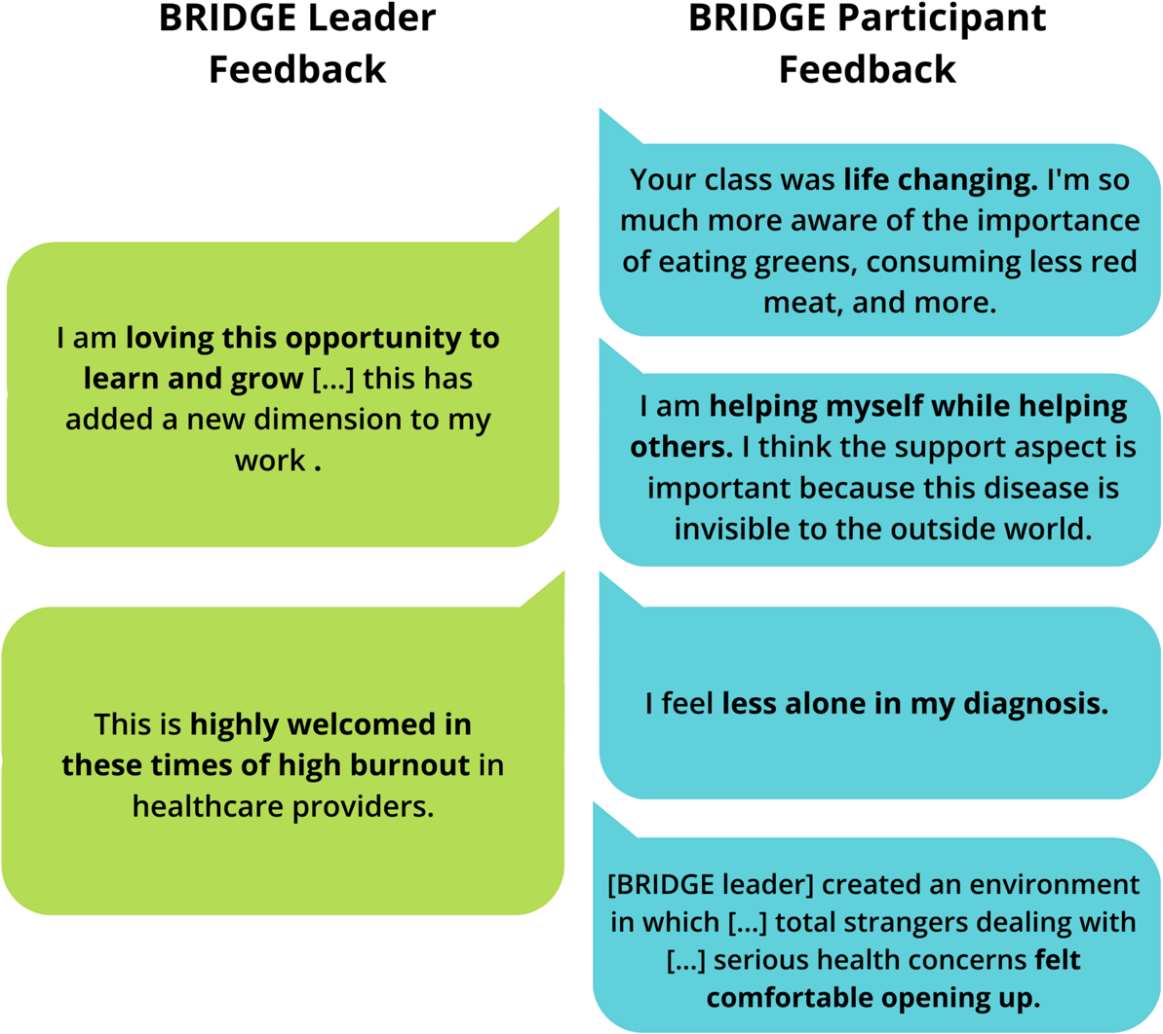
BRIDGE participant and leader feedback regarding program benefits

Patient feedback was obtained after completion of BRIDGE regarding the impact of the program on modifiable behaviors that might impact the course of their MASLD (Fig. 1). Themes of qualitative feedback highlighted perceptions of improved motivation for lifestyle modifications conducive to healthier eating habits, weight loss, and increased in knowledge about MASLD. Patients also subjectively reported feeling less anxiety, distress, and social isolation related to their diagnosis after participating in BRIDGE. Furthermore, patients and providers alike reported significant satisfaction with program participation. Notably, several remarked on the community aspect of the program and its impact on personal views towards weight loss (for participants) or professional identity (as a provider).

We developed standardized documentation for SMAs that complied with requirements for billing, coding, and telemedicine. BRIDGE was initially billed with CPT code 99215 (established patient office visit 40 min) with each participant’s insurance being billed for each visit. Due to national changes in documentation and coding requirements that went into effect in January 2021, documentation was adjusted to reflect time-based billing and included a discussion of medical decision making. We used CPT code 99213, which reflected the total time spent with each individual patient and the time it took to prepare and complete visit documentation; the addition of a 1:1 component was added based on patient requests and served to maintain compliance with billing requirements.

Our plans for ongoing sustainability have been focused on streamlining referrals to the group program, simplifying the workflow of the MAs in scheduling shared medical appointments, and engaging with other stakeholders at our medical institution to share group cohorts. Referrals are being streamlined by directly routing a patient via a smartphrase in the after visit summary to add that patient to the BRIDGE waiting list. The MA workflow has been adjusted to send out invitations via an electronic health record message 3–4 weeks prior to session start, to confirm participation by phone, and for an APP to verbally perform group confidentiality consent if a patient is unable to sign and return a physical or electronic form. Finally, BRIDGE has been disseminated at grand rounds as well as in meetings with clinical leadership for APPs and in other departments, particularly weight management and endocrinology. Our goal is to create a sustainable interdepartmental program that allows for a multidisciplinary approach (i.e. sessions led by clinicians from different divisions) as well as a longer program duration.

## Discussion

Our work demonstrates an APP-led group telehealth SMA program delivered longitudinally is a feasible intervention that fills an important gap in the management of MASLD by leveraging the role of the APP to increase access to health education and behavioral counseling. Notably, we found that telehealth interventions could be offered to older patients with high levels of participation, despite perceived barriers to use of technology. Additionally, the BRIDGE program was accessible to patients with both public and private insurance. Further work is required to determine the program’s long-term impact on sustained weight loss and cardiometabolic outcomes.

Our experience is consistent with prior work demonstrating that group psychoeducational programs promote social learning, offer protection and growth by contributing to others, and foster a safe environment for patients to speak openly, reducing stigma and building hope and confidence. These factors all play an important role in behavior modification [17, 19–21]. Our program emphasized small steps to encourage building of self-efficacy. Behavioral shifts of patients were highly individualized and dependent on readiness for change. Even among those who were not yet ready for behavior change, participants noted that this program improved self-awareness about health behaviors, increased social support among others with a similar diagnosis of liver disease, and facilitated discussion about healthier dietary choices.

Patient acceptance of our group intervention has been favorable, with positive feedback expressed regarding control over health, acquisition of skills to lead to behavior change, social connections, and group support of individualized goals. Some participants have expressed a desire to remain in contact with each other between visits or beyond the BRIDGE group series and voluntarily exchanged contact information. Future work could measure changes in perception of social support, the durability of these social connections, and how to foster these communities outside the realm of discrete group telehealth visits. Further study is needed to better identify areas of highest self-efficacy related to lifestyle changes for MASLD.

Unfortunately, significant social and systemic barriers to patient participation exist, as evidenced by our observation that women and individuals who identified as Hispanic had lower rates of program completion. Language itself was not a barrier, as we were able to translate the content and deliver the program in Spanish by our Spanish-speaking APP. Because prior studies have demonstrated that Hispanic individuals have disproportionally high rates of MASLD and within MASLD are more likely to have more severe disease/fibrosis [48], it is imperative to develop strategies to overcome barriers to accessing programs such as ours. For example, for those with family (i.e., childcare, household chores etc.) and/or work commitments, the program schedule may need adjustment to be in line with the patient’s availability.

It was encouraging to observe that insurance type did not have an impact on program completion; however, given our internal data and patient feedback regarding co-pays associated with our intervention, we advocate for improved insurance coverage to serve the populations most impacted by MASLD. Some state-based affordable care act insurance plans incurred copays for BRIDGE sessions, as well as some employee-based commercial insurance carriers. Patients covered by Medicaid and Medicare as their insurance type did not incur copays. Individuals at high risk of food insecurity, immigrant patients, and publicly insured people may be higher risk populations that need additional financial support to participate.

Telehealth interventions through SMAs such as BRIDGE have the ability to reach a diverse set of patients regardless of patient or provider location and can overcome barriers to health professional shortages, especially in rural areas [49, 50]. However, access to the internet, living in a rural community, lower income, lower educational status, disabilities, and black race or Hispanic ethnicity are all factors that limit access to care via telemedicine [29, 32, 49, 51–53]. We did observe that some patients who had been referred to BRIDGE were unable to participate due inability to access internet or lack of smartphone or computer. Hospital and/or community access points for internet access for video conferencing would be valuable to broaden the reach of BRIDGE and other forms of healthcare delivered via telehealth.

Our intervention was exploratory and brief, and further studies are needed to determine if additional sessions for BRIDGE would be beneficial. A meta-analysis of 11 weight loss interventions found that the best predictors of sustained weight loss were group programs employing MI with a duration of at least 6 months [16], and other weight loss intervention programs programs derived from DPP have an extended duration beyond 6–12 months [24, 27–29]. As the BRIDGE program is intended to promote lifestyle modifications with improved nutrition and increased physical activity, weight loss is likely to happen more gradually but ideally be sustained as the lifestyle changes become incorporated into daily routines of the patients. In our qualitative experience from patient testimonials, BRIDGE participants also view that sessions beyond 12 weeks would be beneficial. Some participants requested an ongoing program as they appreciated the supportive community and the feeling of being accountable to the other group participants.

The SMA care delivery model has the potential to offer additional support to patients with chronic conditions in which health behavior change is the cornerstone of management. SMAs for patients with diabetes have shown the most robust evidence on improvement in glycemic control, diabetes distress and self-care [30, 54] SMAs for weight loss have been shown to result in statistically and clinically significant weight loss, however given the heterogeneity of the interventions and methodological limitations, no strong conclusions could be made on the superiority of SMAs to standard medical care [55]. More research is needed to measure patient reported outcomes of SMAs in comparison to standard medical care. Currently, we have an ongoing follow-up study in progress with qualitative data being collected, including pre- and post-intervention surveys to evaluate for change in knowledge, importance and confidence around making health behavioral changes on a 5-point scale survey, mental and social health changes using the Patient-Reported Outcome Measurement Information System, and the number of minutes of exercise completed per week.

The BRIDGE platform is easily adaptable for liver transplant recipients with MASLD and patients with cirrhosis. The BRIDGE SMA platform may be used as the backbone of supplemental care in patients with alcohol associated liver disease, cirrhosis management, and care for other organ transplant recipients.

## Conclusions

The BRIDGE group telehealth SMA was feasible and acceptable to patients and APPs in a Hepatology clinic setting, with good attendance and positive feedback. Future efforts will focus on optimizing the program design and improving accessibility to patients. Future research is needed to measure the impact of this program on patient-reported outcomes, sustained weight loss and cardiometabolic health outcomes.

## Data Availability

The datasets generated and/or analyzed are not publicly available due to confidential personal health information, but are available from the corresponding author on reasonable request.

## Abbreviations

MASLD: Metabolic dysfunction-associated steatotic liver disease
APP: Advanced Practice Provider
BRIDGE: Behavioral Resources and Intervention through Digital Group Education
SMA: Shared medical appointment
IQR: Interquartile range
USPSTF: US Preventative Services Task Force
MI: Motivational interviewing
DPP: Diabetes Prevention Program
UCSF: University of California at San Francisco
NPs: Nurse Practitioners
PA: Physician Assistant
MAs: Medical Assistants
